# Deletion of *Meg8*-DMR Enhances Migration and Invasion of MLTC-1 Depending on the CTCF Binding Sites

**DOI:** 10.3390/ijms23158828

**Published:** 2022-08-08

**Authors:** Xiao Han, Hongjuan He, Lan Shao, Shuang Cui, Haoran Yu, Ximeijia Zhang, Qiong Wu

**Affiliations:** 1 School of Life Science and Technology, Harbin Institute of Technology, Harbin 150001, China; 2 State Key Laboratory of Urban Water Resource and Environment, Harbin Institute of Technology, Harbin 150001, China

**Keywords:** *Meg8*-DMR, *Dlk1-Dio3* domain, CTCF, Dlk1, MLTC-1, invasion, migration

## Abstract

The *Dlk1-Dio3* imprinted domain on mouse chromosome 12 contains three well-characterized paternally methylated differentially methylated regions (DMRs): IG-DMR, *Gtl2*-DMR, and *Dlk1*-DMR. These DMRs control the expression of many genes involved in embryonic development, inherited diseases, and human cancer in this domain. The first maternal methylation DMR discovered in this domain was the *Meg8*-DMR, the targets and biological function of which are still unknown. Here, using an enhancer-blocking assay, we first dissected the functional parts of the *Meg8*-DMR and showed that its insulator activity is dependent on the CCCTC-binding factor (CTCF) in MLTC-1. Results from RNA-seq showed that the deletion of the *Meg8*-DMR and its compartment CTCF binding sites, but not GGCG repeats, lead to the downregulation of numerous genes on chromosome 12, in particular the drastically reduced expression of *Dlk1* and *Rtl1* in the *Dlk1-Dio3* domain, while differentially expressed genes are enriched in the MAPK pathway. In vitro assays revealed that the deletion of the *Meg8*-DMR and CTCF binding sites enhances cell migration and invasion by decreasing Dlk1 and activating the Notch1-Rhoc-MAPK/ERK pathway. These findings enhance research into gene regulation in the *Dlk1-Dio3* domain by indicating that the *Meg8*-DMR functions as a long-range regulatory element which is dependent on CTCF binding sites and affects multiple genes in this domain.

## 1. Introduction

Genome imprinting is a classic epigenetic phenomenon in which some genes are expressed in a parental-origin-specific manner from just one of the two parental chromosomes [1]. To date, approximately 200 imprinted genes have been discovered in mammals [2]. In mice, by utilizing the “homologous recombination” technique, many studies have proved that imprinted genes play important roles not only in the growth of the embryo, placenta and neonate, but also in the regulation of behavior and neurological abnormalities [3]. The disorder of imprinted genes also leads to human diseases such as cancers and some syndromes including Prader–Willi and Angelman syndromes, Silver–Russell syndrome, Beckwith–Wiedemann syndrome, and Albright hereditary osteodystrophy and Uniparental disomy 14 [4].

The majority of imprinted genes are found in clusters, with each cluster being governed by a single imprinting control region (ICR) [5]. ICRs are typically coincident with germline differentially methylated regions (DMRs), which are locations where maternally and paternally derived alleles have distinct methylation statuses. Most of these ICRs acquire methylation in the female germline and are unmethylated in the male germline; only three regions, *Igf2-H19*, *Dlk1-Dio3*, and *Rasgrf1*, acquire methylation during spermatogenesis [6].

Being located on the distal of chromosome 12, the *Dlk1-Dio3* imprinted domain contains three paternally expressed protein coding genes (*Dlk1*, *Rtl1*, and *Dio3*) and a series of maternally expressed non-coding genes (such as lncRNA *Gtl2*, *Rian, Mirg*, some miRNAs, and snoRNAs) [7,8,9]. In this domain, three paternally methylated DMRs have been well-researched. The most important is IG-DMR, located between *Dlk1* and *Gtl2*, which is a germline DMR and has been proved to be a control element in this region in knockout mice. When it was deleted on a maternally inherited chromosome, maternal to paternal epigenotype transition was detected [10]. The other two DMRs are secondary somatic DMRs: *Gtl2*-DMR (*Meg3*-DMR), which flanks *Gtl2*′s 5′ end [11], and *Dlk1*-DMR, which is found in exon 5 of *Dlk1* [12].

The allelic-specific binding of the CTCF has been identified in the *Meg3*-DMR and demonstrated to be important in the organization of sub-topologically associating domains (TADs) [13,14]. The CTCF insulator protein is enriched on the boundaries of TADs, which are 3D structures with abundant intra-domain connections, protecting genes and their regulatory components from one another [15]. Existing research recognizes the important role played by the CTCF in the regulation of gene expression in some imprinted domains [16]. It was discovered that a subset of gDMRs displayed the allelic recruitment of the CTCF [17]. In the typical *Igf2-H19* domain, the CTCF binds to the unmethylated maternal allele of the *H19*-ICR, which functions as an insulator and controls the imprinted gene expression in this domain [18,19]. Furthermore, in one study, it was found that the insulator activity of the *H19*-ICR varies by cell type in mouse mutant models [20].

The first maternally methylated DMR in the *Dlk1-Dio3* domain, named *Meg8*-DMR, was discovered in the second intron of *Rian* and acquired differential methylation prior to E7.5 [21]. It has been shown that the histone modifications H3K4me3 and H3K9me3 are both abundant in the *Meg8*-DMR, and that the CTCF binds to it non-allele-specifically in vivo [21]. However, the function of *Meg8*-DMR and the targets that it can regulate are unknown.

In this study, first, we demonstrated that the *Meg8*-DMR exhibits insulator activity that is dependent on CTCF binding sites. Then, we dissected the functions of *Meg8*-DMR and its components using the MLTC-1 cell line as a model. The superb down expression of genes on chromosome 12 was detected in the absence of the *Meg8*-DMR, as well as CTCF binding sites, especially the paternal genes *Dlk1* and *Rtl1* in the *Dlk1-Dio3* domain. Furthermore, in MLTC-1, the deletion of the *Meg8*-DMR and CTCF binding sites increased cell migration and invasion and suppressed cell proliferation. When we overexpressed Dlk1 in *Meg8*-DMR-KO, cell invasion and migration were inhibited as the Notch1-Rhoc-MAPK/ERK signaling pathway was blocked. These findings imply that the *Meg8*-DMR is a long-range regulatory element that is dependent on CTCF binding sites and regulates several genes in the *Dlk1-Dio3* domain.

## 2. Results

### 2.1. Meg8-DMR Acts as an Insulator Dependent on the CTCF Binding Sites

**Figure 1:**
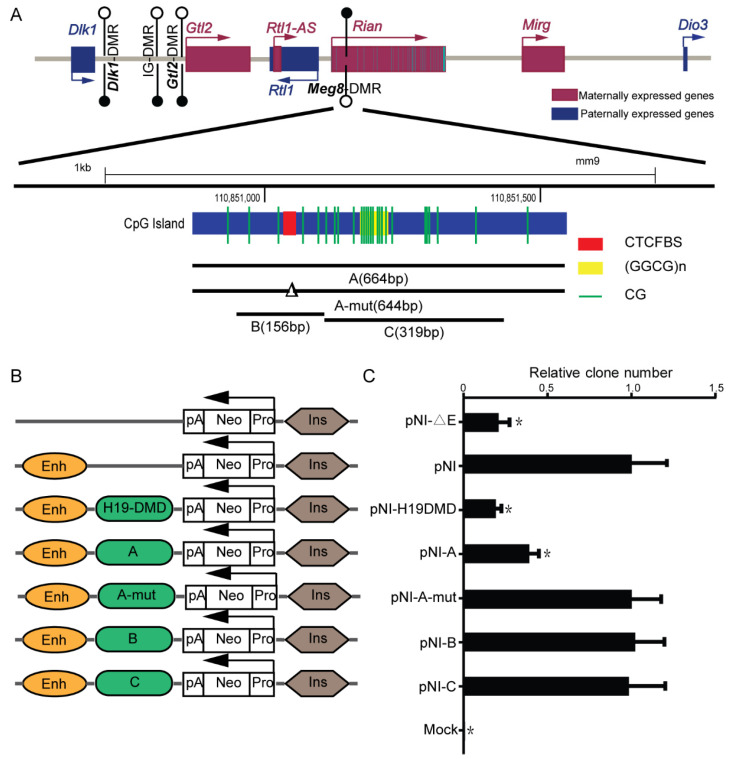
*Meg8*-DMR acts as an insulator dependent on the CTCF binding sites. (**A**) Schematic representation of the *Dlk1-Dio3* imprinted domain. Maternally expressed noncoding RNAs are shown in red, whereas, protein coding paternally expressed RNAs are in blue. Black and white circles represent methylated and unmethylated. *Meg8*-DMR, which is methylated in the maternally inherited chromosome, but unmethylated in the paternally inherited chromosome, is located on the second intron of *Rian* and contains conserved CTCF-binding sites and GGCG repeats. (**B**) The constructs of the enhancer-blocking assay were prepared and transfected into MLTC-1 cells. Each construct contained a mouse β-globin 3′HS2 enhancer element and a human Aγ-globin promoter directing transcription of a neo reporter (Neo), and various test fragments were inserted between them. (**C**) The colony number for each test is expressed relative to the number observed with the control plasmid (PNI). Mock is the blank control group. Error bars, mean ± SEM. *n* = 3. p values were calculated using *t*-test. * *p* < 0.05.

The *Meg8*-DMR, which is located in the second intron of *Rian*, contains conserved CTCF binding sites along with GGCG repeats (Figure 1A). Many known DMRs that bind to the CTCF, such as *H19*-ICR, have the enhancer-blocking properties of an insulator, which is one of the classic mechanisms which explain genomic imprinting [22]. To investigate whether the *Meg8*-DMR also has insulator activity, we used an enhancer-blocking assay, as described previously [23]. The MLTC-1 cell line is a suitable transfection host, and expression of the main genes in the *Dlk1-Dio3* domain, such as *Dlk1*, *Rtl1*, *Gtl2*, *Rian*, and *Mirg*, can be detected. Furthermore, CTCF can bind to CTCF binding sites in the *Meg8*-DMR of MLTC-1 (Figure S1). As a result, we chose the MLTC-1 as a model for our assays. We generated constructs (PNI) in which fragments A–E (Figure 1A) could be inserted between the enhancer and promoter that was driving the neo gene (Figure 1B).

The constructs were introduced into MLTC-1 cells. The clones were counted after four weeks of treatment with G418. When comparing the PNI with a construct without the enhancer, it was shown that the number of clones was reduced by 80%, similar to the previously characterized 1.6kb *H19*-DMD insulator [18] (Figure 1C). The presence of enhancer-blocking activity in the *Meg8*-DMR was confirmed by inserting segment A between the enhancer and the promoter, which reduced the number of clones by 60% (Figure 1C). Next, we removed the 20bp CTCF binding motif in fragment A and obtained a similar clone number to that of the PNI (Figure 1C), indicating that fragment A-mut lost enhancer-blocking activity. Based on these results, we concluded that the *Meg8*-DMR has an insulator function that is dependent on CTCF binding sites.

We also investigated segment B, which only included CTCF binding sites; however, the number of colonies was not considerably reduced (Figure 1C). This demonstrates that *Meg*8-DMR’s insulator activity is influenced by factors other than CTCF binding sites. Furthermore, the GGCG repeats were shown to be insufficient but necessary as fragment C also lacked the insulator function (Figure 1C).

### 2.2. Deletion of Meg8-DMR Reduced the Expression of Many Genes on Chromosome 12

**Figure 2:**
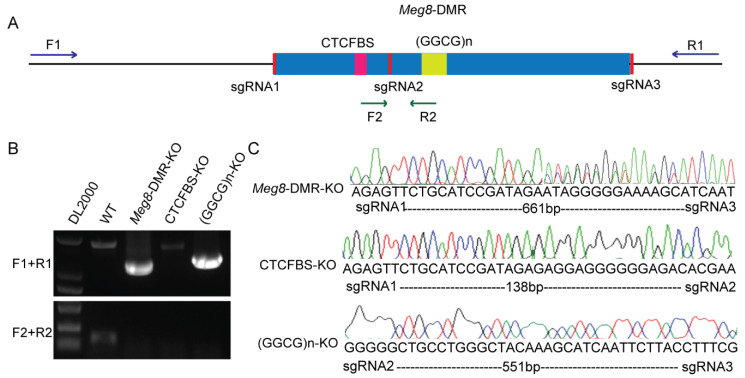
Generation of *Meg8*-DMR knockout cell line models via CRISPR/Cas9. (**A**) A schematic of CRISPR/Cas9-mediated deletion of *Meg8*-DMR, CTCFBS, and (GGCG)n. (**B**) PCR identified homozygous clones, F1/R1primers were located outside of *Meg8*-DMR, and F2/R2 primers were located inside of *Meg8*-DMR. (**C**) The deletion clones of *Meg8*-DMR, CTCFBS and (GGCG)n were identified through sequencing results (*Meg8*-DMR-KO has double peaks close to the targets because the different copies of the chromosomes may be repaired in various ways).

To investigate the target(s) and function of the *Meg8*-DMR, we generated cell line models that knocked out the *Meg8*-DMR and its predicted functional elements using the CRISPR/Cas9 system. Three sgRNAs were synthesized and inserted in a pairwise combinatorial manner into a modified PX458 vector [24], targeting the full-length *Meg8*-DMR, CTCF-binding sites (CTCFBS) and GGCG repeats ((GGCG)n) (Figure 2A). Then, they were independently transferred into MLTC-1 cells. Next, using limiting dilution, we were able to obtain monoclonal cells. The genomic DNA isolated from the clones was then amplified using the outer and inner primers, resulting in the identification of three homozygous cell lines (Figure 2B). The peaks on the chromatogram from DNA sequencing revealed that these three clones were correctly deleted versions of the *Meg8*-DMR, CTCFBS, and (GGCG)n, and they were given the names *Meg8*-DMR-KO, CTCFBS-KO, and (GGCG)n-KO, respectively (Figure 2C).

**Figure 3:**
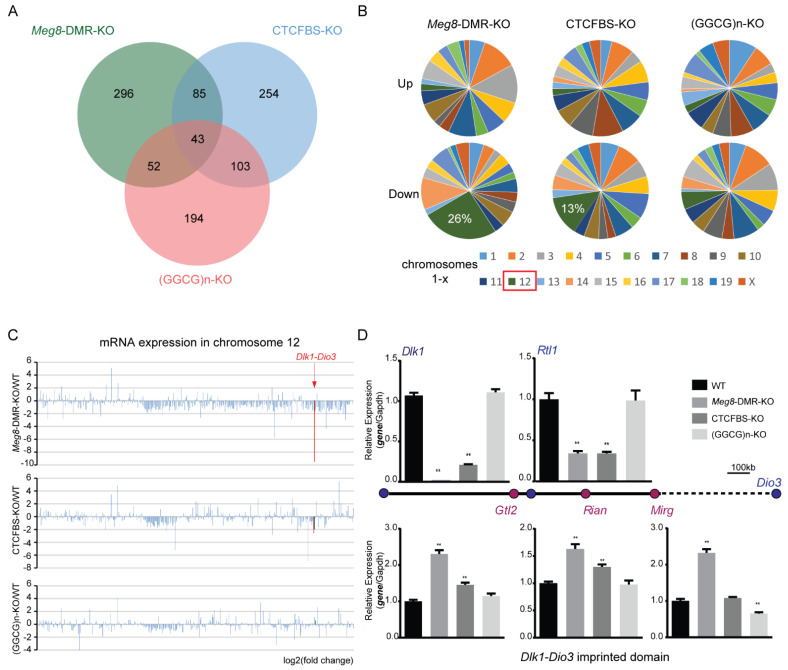
RNA−seq analysis of the *Meg8*−DMR deletion cell lines. (**A**) Venn diagram of upregulated genes and downregulated genes in *Meg8*-DMR-KO, CTCFBS-KO, and (GGCG)n-KO. (**B**) Distribution of upregulated genes and downregulated genes in *Meg8*-DMR-KO, CTCFBS-KO, and (GGCG)n-KO on chromosomes. (**C**) RNA-seq demonstrates log-fold changes of mRNA expression on chromosome 12 in *Meg*8-DMR-KO, CTCFBS-KO, and (GGCG)n-KO compared with WT. Significantly reduced expression of *Dlk1* and *Rtl1* is observed in *Meg8*-DMR-KO and CTCFBS-KO. *Dlk1* and *Rtl1* were marked in red. (**D**) RT-qPCR shows a dramatic reduction in paternal *Dlk1* expression and slightly increased expression of maternal *Gtl2*, *Rian*, and *Mirg* lncRNAs in *Meg8*-DMR-KO and CTCFBS-KO compared with WT. Strand-specific RT-PCR shows a dramatic reduction in paternal *Rtl1* expression in *Meg8*-DMR-KO and CTCFBS-KO compared with WT. The relative expression level was normalized by *Gapdh*. Error bars, mean ± SEM. *n* ≥ 3. *p* values were calculated using *t*-test. ** *p* < 0.01.

To learn more about the involvement of the *Meg8*-DMR in MLTC-1 gene regulation, we used high-throughput sequencing to profile mRNA expression in *Meg8*-DMR-KO, CTCFBS-KO, (GGCG)n-KO, and WT of MLTC-1. In comparison to the WT, these three cell lines had 476, 485, and 392 differentially expressed genes (DEGs), respectively (Figure 3A). In the *Meg8*-DMR-KO and CTCFBS-KO cell lines, the majority of the down-regulated genes were found on chromosome 12; however, in the (GGCG)n-KO cell line, the genes were evenly distributed across all chromosomes (Figure 3B). Then, we looked at all the genes detected on chromosome 12, where the *Meg8*-DMR was discovered. In addition to the DEGs, there were a large number of other genes with decreased expression in the *Meg8*-DMR-KO and CTCFBS-KO cell lines, particularly those in and near the *Dlk1-Dio3* imprinted domain (Figure 3C). Furthermore, RT-qPCR analysis revealed a significant reduction in the expression of the paternally inherited protein coding gene *Dlk1*, while maternally expressed lncRNAs (*Gtl2*, *Rian*, and *Mirg*) were up-regulated in *Meg8*-DMR-KO and CTCFBS-KO compared with WT (Figure 3D). Similarly, strand-specific RT-PCR analysis also showed a significant reduction in the expression of the paternally inherited protein coding gene *Rtl1* in *Meg8*-DMR-KO and CTCFBS-KO. Additionally, the expression of these genes in (GGCG)n-KO was comparable to that in WT (Figure 3D).

**Figure 4:**
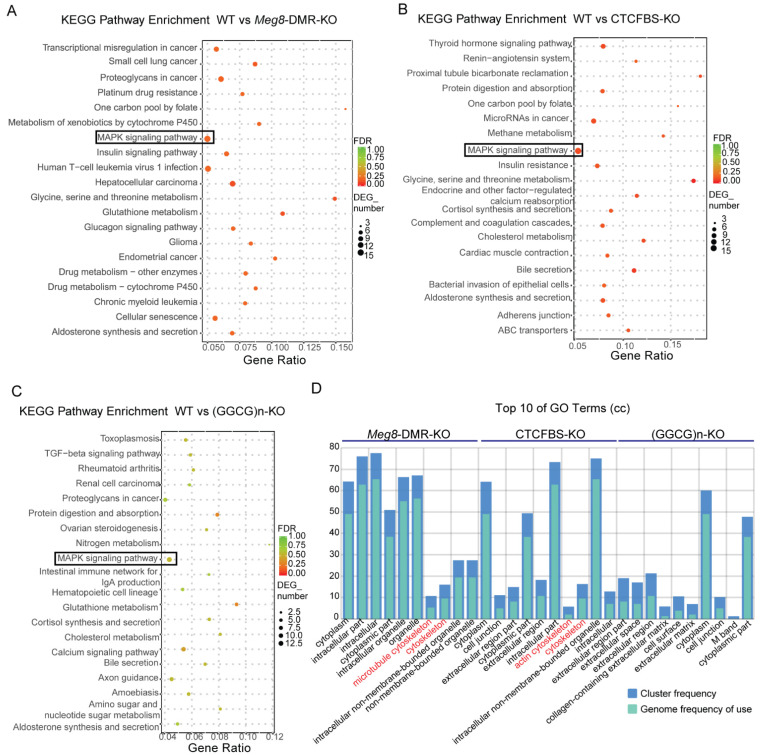
DEGs Functional Analysis of the *Meg8*-DMR deletion cell lines. (**A**–**C**) Top 20 of KEGG pathway enrichment of genes differentially expressed in *Meg8*-DMR-KO, CTCFBS-KO, and (GGCG)n-KO. (**D**) Top 10 GO terms of cellular component (CC) of GO enrichment analysis of differentially expressed genes in *Meg8*-DMR-KO, CTCFBS-KO and (GGCG)n-KO.

To further understand the effect of the *Meg8*-DMR and its component deficiencies in MLTC-1, we compared the DEGs of *Meg8*-DMR-KO, CTCFBS-KO, and (GGCG)n-KO to WT using KEGG pathway enrichment analysis and gene ontology (GO) enrichment analysis. The MAPK pathway was enriched in all three cell lines, and it was more significant in *Meg8*-DMR-KO and CTCFBS-KO than in (GGCG)n-KO, according to the KEGG pathway enrichment analysis (Figure 4A–C). The GO enrichment analysis revealed that the cellular component was enriched in the cytoskeleton of *Meg8*-DMR-KO and CTCFBS-KO but not in ((GGCG)n-KO (Figure 4D).

### 2.3. Meg8-DMR Deletion Enhanced Migration and Invasion of MLTC-1

**Figure 5:**
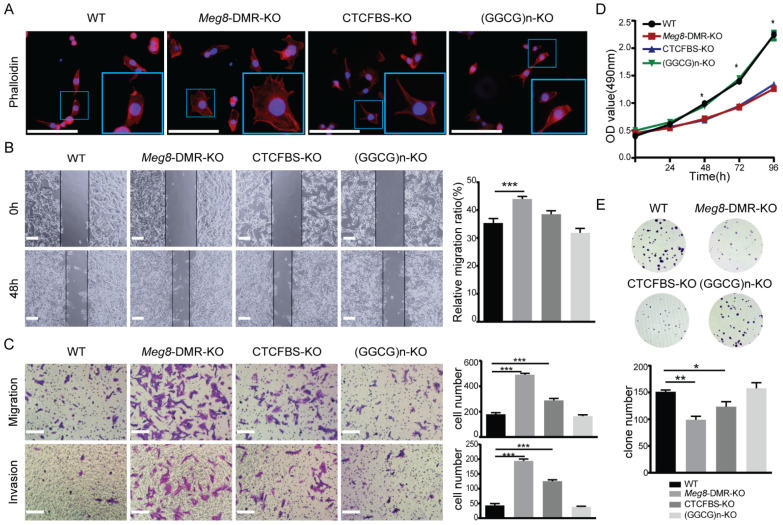
*Meg8*-DMR deletion enhanced migration and invasion of MLTC-1. (**A**) Immunofluorescence staining of Phalloidin-California Red Conjugate in WT, *Meg8*-DMR-KO, CTCFBS-KO, and (GGCG)n-KO. (**B**) Confluent monolayers of four cell lines were wounded and after being incubated for an additional 48 h, the relative migration ratio was calculated. (**C**) Migration and invasion of four cell lines were measured via Transwell assay. (**D**) Cell proliferation of four cell lines was measured via MTT assay. (**E**) Colonies grown from cells of four cell lines were counted. Error bars, mean ± SEM. *n* ≥ 3. *p* values were calculated using *t*-test. * *p* < 0.05, ** *p* < 0.01, *** *p* < 0.001, scale bars = 200 μm.

In order to understand the biological function of *Meg8*-DMR, we utilized various assays in vitro. According to the results of GO analysis, we initially detected the impact of the deletion of *Meg8*-DMR on the actin cytoskeleton in MLTC-1 cells by examining the Phalloidin-California Red Conjugate staining of F-actin. The results showed that the cytoskeleton distribution of *Meg8*-DMR-KO and CTCFBS-KO changed, with filopodia in *Meg8*-DMR-KO being noticeably more abundant, and filopodia in CTCFBS-KO also being more plentiful than those in WT and (GGCG)n-KO (Figure 5A). The mobility of cells was then examined based on this outcome. In comparison with WT, the migratory speed in *Meg8*-DMR-KO was much higher, whereas the speed in CTCFBS-KO was also higher but not significant, and the speed in (GGCG)n-KO was essentially unchanged (Figure 5B). Similarly, Transwell analysis revealed that *Meg8*-DMR-KO and CTCFBS-KO had greater abilities to migrate and invade than WT and (GGCG)n-KO, and that the change in *Meg8*-DMR-KO was more significant than that in CTCFBS-KO (Figure 5C).

Furthermore, we explored the effects of *Meg8*-DMR deletion on cell growth using the MTT assay. Interestingly, *Meg8*-DMR and CTCFBS deletion dramatically decreased cell growth at 72 and 96 h, despite the shift in cell mobility (Figure 5D). The clone creation assay was then utilized to confirm the effects of the *Meg8*-DMR on proliferation. Consistent with the MTT experiment, the data demonstrated that the efficiency of colony formation in *Meg8*-DMR-KO and CTCFBS-KO was greatly reduced (Figure 5E). Both assays indicated that deleting (GGCG)n had no effect on cell proliferation (Figure 5D,E).

### 2.4. Overexpression of Dlk1 in Meg8-DMR-KO Suppressed Cells’ Migration and Invasion by Blocking Notch1-Rhoc-MAPK/ERK

**Figure 6:**
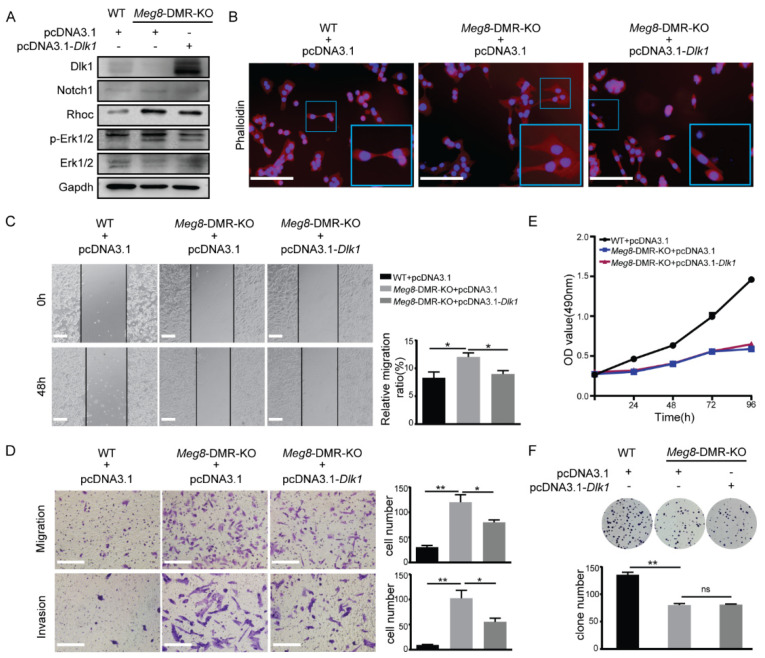
Overexpression of Dlk1 in *Meg8*−DMR−KO suppressed cells’ migration and invasion by blocking Notch1−Rhoc−MAPK/ERK. (**A**) Western blot shows the expression level of Dlk1 was reduced while Nocth1 Rhoc and p-ERK1/2 were activated in *Meg8*-DMR-KO. When the overexpression vector pcDNA3.1-*Dlk1* was transfected into *Meg8*-DMR-KO, the expression of Nocth1, Rhoc, and p-ERK1/2 was reduced. (**B**) Immunofluorescence staining of Phalloidin-California Red Conjugate in WT, *Meg8*-DMR-KO, and *Meg8*-DMR-KO with Dlk1 overexpression. (**C**) Confluent monolayers of cells of WT, *Meg8*-DMR-KO and *Meg8*-DMR-KO with Dlk1 overexpression were wounded and after being incubated for an additional 48 h, the relative migration ratio was calculated. (**D**) Migration and invasion of WT, *Meg8*-DMR-KO, and *Meg8*-DMR-KO with Dlk1 overexpression were analyzed. (**E**) Cell proliferation of WT, *Meg8*-DMR-KO and *Meg8*-DMR-KO with Dlk1 overexpression was measured via MTT assay. (**F**) Colonies grown from cells of WT, *Meg8*-DMR-KO, and Meg8-DMR-KO with Dlk1 overexpression were counted. Error bars, mean ± SEM. *n* ≥ 3. *p* values were calculated using *t*-test. * *p* < 0.05, ** *p* < 0.01, ns (non-significant), scale bars = 200 μm.

We attempted to rescue Dlk1 expression in the *Meg8*-DMR-KO clone to study the mechanisms by which *Meg8*-DMR loss can lead to alterations in MLTC-1 cells, as *Dlk1* is one of the most significantly changed genes in the deletion of the *Meg8*-DMR. The Western blot results demonstrated that the overexpression of Dlk1 was achieved after transfecting pcDNA3.1-*Dlk1* into *Meg8*-DMR-KO (Figure 6A). The exchange of *Meg8*-DMR-KO was then observed after Dlk1 was overexpressed. First, by examining the Phalloidin-California Red Conjugate staining of F-actin, we discovered that the overexpression of Dlk1 repaired the cytoskeleton distribution alteration caused by *Meg8*-DMR deletion (Figure 6B). Furthermore, when Dlk1 was overexpressed, the migratory speed of *Meg8*-DMR-KO was reduced in a wound-healing assay (Figure 6C). Similarly, in Transwell assays, we found that overexpressing Dlk1 inhibited *Meg8*-DMR-KO migration and invasion (Figure 6D). We then tested some proteins that may have contributed to this process in order to thoroughly examine its molecular underpinnings. According to many studies, Dlk1 is identified as a Notch family protein and the best studied noncanonical Notch ligand [25,26], so we first tested Notch1, one of the four Notch receptors, which has been verified to directly interact with Dlk1 in both yeast and mammalian two-hybrid systems [27,28]. Then, we looked at Rhoc, which is a protein that plays a key role in cell migration [29]. Taking the results of the KEGG analysis, we also tested the Erk1/2. Notch1, Rhoc, and p-Erk1/2 were increased when *Meg8*-DMR was deleted, and they decreased when Dlk1 was overexpressed in *Meg8*-DMR-KO (Figure 6A). In conclusion, the deletion of the *Meg8*-DMR decreased Dlk1 and increased MLTC-1 migration and invasion via Notch1-Rhoc-MAPK/ERK.

We also used MTT and colony formation assays to determine whether Dlk1 overexpression had any influence on cell proliferation, and the results demonstrate that it had no effect in *Meg8*-DMR-KO (Figure 6E,F).

### 2.5. Absence of Meg8-DMR Has No Impact on the Methylation of Other DMRs in the Dlk1-Dio3 Domain

Including the IG-DMR, which serves as the imprinting control region, there are three known paternally methylated DMRs which can regulate the expression of imprinted genes in the *Dlk1-Dio3* domain [30]. We employed bisulfite sequencing to examine DNA methylation in WT, *Meg8*-DMR-KO, CTCFBS-KO, and (GGCG)n-KO cell lines to determine whether the deletion of the *Meg8*-DMR can affect the methylation status of these three DMRs.

**Figure 7:**
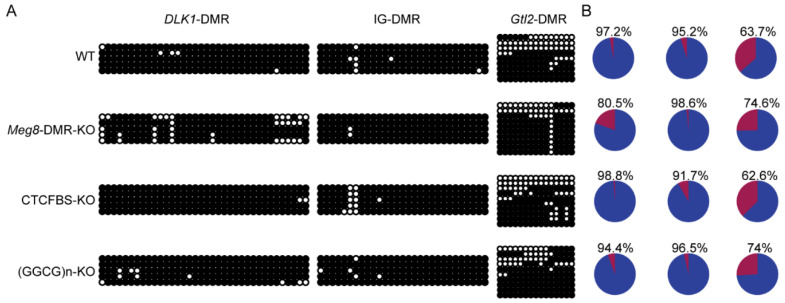
Methylation of the *Dlk1-Dio3* domain in the absence of *Meg8*-DMR. (**A**) Bisulfite sequencing analysis of *Dlk1*-DMR, *IG-DMR*, and *Gtl2*-DMR in each cell line. Each CpG dinucleotide is represented with a circle. Each row of circles represents an individual clone sequenced. Black and white circles represent methylated and unmethylated CpGs, respectively. (**B**) Percentage statistics of methylation status.

The results showed that the *Dlk1*-DMR and IG-DMR were hypermethylated in the WT MLTC-1 cell line, but the *Gtl2*-DMR was hypomethylated in one half and hypermethylated in the other half (Figure 7A). The methylation status of these three DMRs in the examined cell lines and WT were not significantly different (Figure 7A). This implies that the absence of the *Meg8*-DMR does not affect the methylation of *Dlk1*-DMR, IG-DMR, and *Gtl2*-DMR and in the present case, the deleted *Meg8*-DMR itself may have contributed to the regulation of gene expression.

## 3. Discussion

Numerous studies have emphasized the role of imprinted genes in the control of embryonic growth, development, and the etiology of major disorders since their discovery in the 1980s [31]. *Dlk1-Dio3*, like *Igf2-H19*, is an imprinted domain in which both maternal and paternal uniparental disomies cause fetal mortality, and it also acts as a parthenogenesis blocker [32,33]. This suggests that the imprinted genes in this region are crucial for growth and development. The important functions of various genes in the *Dlk1-Dio3* domain in mammalian development and disease incidence have been established using gene knockout models. As a result, a thorough analysis of epigenetic modifications in this region is necessary to understand the regulation of imprinting and gene expression.

In our investigation, we utilized genetic engineering and targeted epigenetic elements editing in MLTC-1 to dissect the regulatory mechanisms of the *Meg8*-DMR, the first discovered maternal DMR that has not been well characterized in the *Dlk1-Dio3* domain. For the first time, our findings describe the genes that the *Meg8*-DMR targets, as well as the phenotypes in MLTC-1 when distinct portions of the *Meg8*-DMR are knocked out. More importantly, our research sheds light on the structure of the *Meg8*-DMR and identifies the critical role played by CTCF binding sites.

The insulator model, which relies on the transcription factor CTCF that bonds in DMRs to regulate imprinted gene expression in a parental-origin-specific manner in some imprinted clusters, is one of the cis-acting silencing mechanisms that are proposed to govern imprinting [34]. In a common scenario, *H19*-DMD, a gDMR in the *H19-Igf2* domain, acts as an insulator, which is described as an element that sits between the enhancer and the promoter and prevents them from interacting, and subsequently serves as the ICR for this region [18,35,36]. This model has also been identified in other DMRs such as the *Rasgrf1*-DMR, KvDMR1, and *Grb10*-DMR [37,38,39]. Using the enhancer blocking experiment in MLTC-1, the *Meg8*-DMR, which contains conserved CTCF binding sites, was shown to function as an insulator as well as the *H19*-DMD. In the *Meg8*-DMR insulator model, the CTCF binding sites are the core element, but this element alone cannot block the enhancer. This means that GGCG repeats are required in this model. Tandem repeats are a common occurrence in imprinting centers [40] and some of them serve as a high frequency of binding sites for transcription factors and participate in the regulation of imprinted gene expression. The tandem repeat in the *H19-IGF2* intergenic DMR concentrates the methylation-restricted binding of the key transcription factors ZFP57 and CTCF [41,42]. In the recombination of tandem repeats, recurring imprinting errors can be seen [43]. Additionally, the tandem repeats in the IG-DMR have also been demonstrated to be useful through deletion model construction [44]. However, some repeats are nonfunctional, such as the long-interspersed elements (LINE-1) in the *DlK1-Dio3* domain, the deletion of which does not disrupt imprinting and does not result in aberrant phenotypes in mutant mice [45].

Through CRISPR knockout experiments, we revealed that the *Meg8*-DMR is a functional element in the regulation of gene expression on chromosome 12, especially in the *Dlk1-Dio3* domain. An identical gene expression pattern can be obtained by removing the entire *Meg8*-DMR or simply the CTCF binding sites. This is in line with the findings from the insulator activity assay, and CTCF binding sites were shown to be important in the *Meg8*-DMR. The cell morphology and behavior assays showed a similar pattern of results. The ablation of the *Meg8*-DMR altered the distribution of F-actin and increased MLTC-1 cell invasion and migration while decreasing proliferation considerably. When the CTCF-binding sites were knocked out, the phenotypes followed a similar pattern, but not to the same extent. Meanwhile, some data suggest GGCG repeats could be a possible supporter of the *Meg8*-DMR, because the deletion of GGCG repeats produces fewer alterations in gene expression and essentially no changes in cell morphology and behavior when compared to the *Meg8*-DMR and CTCF binding sites.

When we rescued Dlk1 expression in the *Meg8*-DMR deletion cell line, the distribution of F-actin was recovered, and the migration and invasion of cells were suppressed compared to the *Meg8*-DMR-KO cells without Dlk1 overexpression. Additionally, Notch1 was reduced company with Rhoc and p-ERK1/2. These results are consistent with the findings of a number of previous studies on other cells. Despite the fact that many contradictory studies from different models suggest that Dlk1 can stimulate, inhibit, or have no effect on Notch signaling, it is widely accepted that Dlk1 acts as a Notch1 inhibitor in some cases [46,47,48,49] via cis-inhibition by competing with conventional ligands [50]. In cervical cancer, Notch1 has been reported to lead to changes in migration and invasion by regulating Rhoc [51] and it has also been found to make similar phenotypic contributions with Rhoc in some other cancers such as non-small-cell lung cancer [52] and ovarian cancer [53]. Rhoc, a member of the RHO GTPase family, has been proven to enhance cancer cells migration, invasion, and metastasis through the regulation of cytoskeletal organization [54]. In highly metastatic inflammatory breast cancer, Rhoc can induce motility and invasion through the activation of the MAPK/ERK pathway [55]. These results reveal that the deletion of the *Meg8*-DMR enhanced the migration and invasion of MLTC-1 through lowering Dlk1 and then activating the Notch1-Rhoc-MAPK/ERK pathway.

However, the cell growth and colony formation of MLTC-1 were not restored when Dlk1 was overexpressed. This effect of deletion of the *Meg8*-DMR on MLTC-1 could be influenced by other genes. Previous research has shown that many genes in *Dlk1-Dio3* have a relationship with cell proliferation. *MEG3*, a human ortholog of *Gtl2*, was considered to be a LncRNA tumor suppresser and its re-expression inhibits tumor cell proliferation and colony formation [56]. The suppression of *RTL1* was found to inhibit the growth of melanoma cells [57]. It has also been reported that increased *MEG8*, a human ortholog of *Rian*, expression inhibited the proliferation of trophoblast and vascular smooth muscle cell lines [58,59], but *MIRG* inhibition markedly reduced the activity of bone marrow macrophages in terms of cell proliferation [60]. For all of these genes that changed after *Meg8*-DMR deletion and which may have effects on the proliferation ability of cells, further research is required to investigate the mechanism by which *Meg8*-DMR deletion affects the cell growth of MLTC-1. Besides, a study examined the role of *MEG8* and *MEG3* in migration and invasion, with both *MEG8* and *MEG3* expression causing epithelial–mesenchymal transition-related cell morphological changes and increased cell motility [61]. Additionally, in BEAS-2B cells, *MEG8* overexpression facilitated cell proliferation, invasion, and migration [62]. The ability of the HCC cells to proliferate, migrate, and invade was significantly suppressed by *MEG8* inhibition [63]. Considering these findings, the relationship between these genes and the cell phenotypes in *Meg8*-DMR mutant cells requires further investigation.

In addition, there are many microRNAs in the *Dlk1-Dio3* domain whose targets can be found on all the chromosomes. We used the ENCORI (https://starbase.sysu.edu.cn/) to analyze the differentially expressed genes which may be the targets of microRNAs. The results are listed in Table S5. In the *Meg8*-DMR-KO cell line 347 of 476 DEGs could be the targets of 80 microRNAs in the *Dlk1-Dio3* domain, and 278 of these genes were outside chromosome 12. For each gene may be the target of multiple microRNAs and each microRNA can also target multiple genes, the correlation between DEGs and microRNAs in the *Dlk1-Dio3* is complex and worth exploring for future research.

According to several studies, the IG-DMR and *Gtl2*-DMR regulate imprinted genes in the *Dlk1-Dio3* domain in a coordinated and long-range manner. When the IG-DMR was deleted on the maternally inherited chromosome, all the genes in the cluster lost imprinting, the expression of maternally expressed noncoding genes was silenced, and the paternally expressed protein coding genes had the opposite expression [10]. The same gene expression pattern silencing of maternally expressed genes and activation of PEGs was also reported in the mat deletion of the *Gtl2*-DMR in mice [64,65]. In *Meg8*-DMR-KO and CTCFBS-KO, the methylation status of these two DMRs was maintained, indicating the direct regulatory function of the *Meg8*-DMR by itself, rather than through the other two DMRs. Although the majority of known differentially methylated regions have regulatory functions in non-methylated alleles [1,66], the *Meg8*-DMR which is methylated (data not shown) in MLTC-1 is still functional. This may because the CTCF binds to this binding site in a biallelic manner by not actually containing CpG dinucleotide [21]. In our study, the necessity of CTCF binding to the *Meg8*-DMR was shown through the insulator activity assay and the deletion of CTCF binding sites, which resulted in the change in gene expression and cell phenotype. More research is needed to determine how the *Meg8*-DMR regulates gene expression in the *Dlk1-Dio3* domain through binding with the CTCF.

## 4. Materials and Methods

### 4.1. Cell Culture

The MLTC-1 (ATCC CRL-2065) cells were cultured in RPIM1640 (GIBCO, Carlsbad, CA, USA) containing 10% fetal bovine serum with penicillin/streptomycin at 37 °C with 5% CO_2_ in a humidified incubator.

### 4.2. The Enhancer Blocking Assay

The enhancer blocking assay was performed as described previously [23]. The human Aγ-globin promoter and a mouse β-globin 3′HS2 enhancer element were used to activate a gene for neomycin resistance in each reporter. 2.5 × 10^5^ cells of MLTC-1 were seeded in each well of 12-well plates; after 12 h, an equal number of moles of each construct was transfected into the MLTC-1 cells in each well using Lipofectamine™ 3000 (#L3000015, Invitrogen, Carlsbad, CA, USA), and cells of one well were not transfected with any constructs as a blank control. Then, 48 h after transfection, 5 × 10^5^ cells of each well were seeded in new dishes and cultured in the medium containing 150 μg/mL G418. After 3–4 weeks, when the cells in the blank control were all dead, these cells were fixed with 4% PFA for 30 min and then stained with 0.1% crystal violet for 30 min. At last, the number of colonies was counted and normalized to that obtained with pNI. An equal number of moles of each construct were transfected into the MLTC-1 cells in order to normalize the copy number among the various mutants. After 4 weeks, the number of colonies that were resistant to G418 was counted. Three independent replications were performed. Primer sequences of the fragments that were inserted in each reporter are listed in Table S1.

### 4.3. CRISPR/Cas9-Mediated Deletion of Meg8-DMR, CTCF Binding Sites and GGCG Repeats

The single-guide RNAs for deletion were designed by using http://crispr.mit.edu/ (accessed on 3 November 2014), and http://crispr.dbcls.jp/ (accessed on 8 September 2016). SgRNAs were annealed with NEBuffer2 for 2 min at 90 °C, 1 h at 37 °C, and finally 1 h at room temperature. *BbsI* and *BsaI* (NEB, Ispawich, MA, USA) were used to insert annealed double-stranded DNA into the CRISPR/Cas9 PX458 vector, which was modified [24]. Then, the purified recombinant plasmids were transfected into MLTC-1 cells in 24-well plates. After being screened using puromycin, the cells were seeded into 96-well plates with limiting dilution. Two weeks later, the DNA of the clones was isolated and then identified via PCR using external and internal primers of *Meg8*-DMR. Primer sequences are listed in Table S1.

### 4.4. DNA Extraction and Methylation Analysis

Each cell line’s genomic DNA was obtained using a conventional procedure that included phenol/chloroform extraction and ethanol precipitation. The isolated DNA was modified with bisulfite according to the manufacturer’s instructions using the EZ DNA Methylation-Gold Kit (Zymo Research, cat# D5005, Irvine, CA, USA). Zymo*Taq*™ DNA Polymerase (Zymo Research, cat#E2001, Irvine, CA, USA) was used to amplify bisulfite-treated materials using a nested PCR. Table S1 lists the primer sequences. The PCR products were cloned into the pMD19T vector. The subclones were then identified and chosen for sequencing.

### 4.5. RNA Extraction and RNA-Seq Analysis

Total RNAs were prepared from each cell line by RNAiso Plus (TaKaRa, Dalian, China) according to the manufacturer’s protocol and then given to Personal Bio Company for transcriptome sequencing. The library was then subjected to Paired-End sequencing on the Illumina Hiseq sequencing technology after it was produced. Raw data were filtered using Cutadapt, and quality control was performed using FastQC. Paired-end Fastq files were mapped to ENSEMBL Mouse assembly GRCm38 (mm10) using Tophat2. DESeq was used to analyze the differentially expressed genes (DEGs), and absolute fold change >2 or <0.5 and *p*-value < 0.05 were used to identify DEGs. All the DEGs of the three cell lines are listed in Tables S2–S4.

### 4.6. qRT-PCR and Strand-Specific RT-PCR

The cDNA was synthesized via reverse transcription using the PrimeScriptTM RT reagent Kit with gDNA Eraser (TaKaRa). Then, the qRT-PCR was performed using TB Green^®^ Premix Ex Taq™ II (Tli RNaseH Plus) (TaKaRa) on an ABI ViiA 7 system. The results were analyzed using the relative quantitative method, and the mRNA expression of genes was normalized with *Gapdh*. The primers for qRT-PCR are shown in Table S1.

A strand-specific reverse transcriptase reaction was performed using the method a previous study reported [67] for the detection of paternally expressed *Rtl1* using the PrimeScriptTM RT reagent Kit with gDNA Eraser (TaKaRa). The PCR results were quantified with Image J 1.53e software (Wayne Rasband and contributors National Institutes of Health, USA, http://imagej.nih.gov/ij), and the results were normalized to *Gapdh* expression.

### 4.7. Immunofluorescence

The cells of each cell line were cultured on 48-well chamber slides and fixed with 4% PFA for 30 min. After being washed three times with PBS, fixed cells were incubated in 0.1% Triton X-100 in PBS for 5 min. Then, the cells were stained with Phalloidin-California Red Conjugate (#B8325, APExBIO) at 37 °C for 1 h. Finally, the slides with cells were incubated with DAPI (Invitrogen).

### 4.8. Wound Healing, Cell Migration and Invasion

For the wound-healing assay, each cell line was cultured into a confluent monolayer and then scratched with a 10 μL pipette tip. Medium, which was refreshed into serum-free, was added to culture cells to close the wounds for 48 h.

5 × 10^4^ cells of each cell line were placed on the top sides of Transwell chambers (Corning, Kennebunk, ME, USA), after incubation for 24 h, while the Transwell chambers were coated with Matrigel (Corning, Bedford, MA, USA) for 48 h, and the lower surface cells were fixed with 4% paraformaldehyde (PFA) at room temperature for 30 min and stained with 0.1% crystal violet for 30 min. Four independent fields were counted under the microscope.

### 4.9. MTT and Colony Formation Assays

3 × 10^3^ cells of each cell line were seeded in each well of 96-well plates. After every 24 h at the indicated time, 20 μL of MTT were added to 200 μL of culture medium per well. After 4 h of incubation at 37 °C, the medium was removed and 100 μL of dimethyl sulfoxide was added. The absorbance was measured at a wavelength of 490 nm in a plate reader (Tecan i-control infinite 200).

3 × 10^2^ cells of each cell line were seeded in each well of 6-well plates, and after being cultured for 3 weeks, these cells were fixed with 4% PFA for 30 min and then stained with 0.1% crystal violet for 30 min, and then the number of colonies was counted.

### 4.10. Western Blotting Analysis

The total protein content was extracted from cells of each cell line by using RIPA lysis buffer and then determined by using the BCA method. The whole protein of each sample was separated using 10% SDS-PAGE and transferred onto PVDF membranes for blotting. The antibodies used in this study were Gapdh (#60004-1-Ig Proteintech, Wuhan, China), Dlk1 (#ab210471, Abcam, Cambridge, UK), Notch1 (#4ab094535, 4A BIOTECH, Beijing, China), Rhoc (#4ab090482, 4A BIOTECH, Beijing, China), Erk1/2 (#BF8004, Affinity Biosciences, Changzhou, China), and p-Erk1/2 (#AF1015, Affinity Biosciences, Changzhou, China). The full length of *Dlk1* cDNA was cloned to pcDNA3.1 to overexpress Dlk1.

## 5. Conclusions

In this study, we demonstrated that the *Meg8*-DMR has insulator activity depending on CTCF-binding sites in MLTC-1. The deletion of the *Meg8*-DMR altered the expression of genes in the *Dlk1-Dio3* imprint domain, which suppressed cell growth and promoted invasion and migration, with CTCF binding sites playing a key role. We preliminarily identified the targets and biology function of the *Meg8*-DMR in MLTC-1; however, more research is needed to understand how it regulates gene expression.

## Data Availability

RNA-seq sequencing data have been deposited in GEO under accession number

## Supplementary Materials

The supporting information can be downloaded at: https://www.mdpi.com/article/10.3390/ijms23158828/s1. Reference [24] is cited in the supplementary materials.
