# Supplementary material for: Deletion of Meg8-DMR Enhances Migration and Invasion of MLTC-1 Depending on the CTCF Binding Sites
Source: Int J Mol Sci. 2022 Aug 8;23(15):8828. doi: 10.3390/ijms23158828 (PMC9369160; doi:10.3390/ijms23158828)
Supplement: Supplementary file 1 [file ijms-23-08828-s001.zip › Figure S1.pdf]

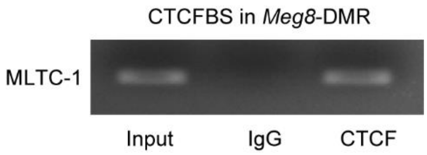


**Figure S1. ChIP analysis of CTCF in CTCFBS in MLTC-1 cells.** The ChIP assay was performed as described before [24]. The primers used for the ChIP analysis are same with the fragment A in the enhancer blocking assay.
