# Supplementary material for: Deletion of Meg8-DMR Enhances Migration and Invasion of MLTC-1 Depending on the CTCF Binding Sites
Source: Int J Mol Sci. 2022 Aug 8;23(15):8828. doi: 10.3390/ijms23158828 (PMC9369160; doi:10.3390/ijms23158828)
Supplement: Supplementary file 1 [file ijms-23-08828-s001.zip › Table S1.pdf]

TableS1 Primer Sequences

Primer name	Primer sequence	
sgRNA1	GTTCTGCATCCGATAGAAGTTGG	
sgRNA2	CCAAATGAGGAGGGGGGAGACAC	
sgRNA3	GGGCGGCTGCAAACAGCGATAGG	Knockout targets and amplification primers for identifying homozygous clones
External primer F1	GTGGGCAAAGGATGGATTGA	
External primer R1	TGGGTGGACTGAAGGGAAAG	
Internal primer F1	TGCCAACTCACGGTGCAGTGGAAAT	
Internal primer F2	TCCAAAGCGTAGGTCCCTCTTCGTGTC	
Fragment A-F	GGATGGGGATAGAGGAGT	
Fragment A -R	ACAGCAAACTGACTCACCTT	
Fragment B-F	CTTAAGATTTGTGCCAACTCAC	
Fragment B-R	GGTCCCTCTTCGTGTCTC	primers for construction of enhancer blocking assay vector
Fragment C-F	TACGCTTTGGATTTCTGCGGCAGC	
Fragment C-R	CAGGCTCTTCTTCACTCTTCC	
Fragment A-mut -F	GGGCTACAGTTTTTGCGTTCCCAAATG	
Fragment A-mut -R	TTGGGGGCGCCACAGACTCTTGGCTGC	
Dlk1-F	ACGGGAAATTCTGCGAAATA	
Dlk1-R	CTTTCCAGAGAACCCAGGTG	
Gtl2-F	CGAGGACTTCACGCACAAC	
Gtl2-R	TTACAGTTGGAGGGTCCTGG	
Rian-F	TAGAGTCTCCCTTGAAAGTGG	
Rian-R	TGGTATCTATAAGAACAGAGCTGA	
Mirg-F	GTTGTCTGTGATGAGTTCGC	
Mirg-R	GTTCTTGAACATCCGCTCC	qRT-PCR Primers
Gapdh-F	GTCGTGGAGTCTACTGGTGTC	
Gapdh-R	GAGCCCTTCCACAATGCCAAA	
Dlk1-DMR-F1	ACATGGCCCTCCCAAGACCT	
Dlk1-DMR-R1	GCTGCAGCCAGCTTGTTCCT	
Dlk1-DMR-F2	AATCGTCCCAGAGTGATG	
Dlk1-DMR-R2	GTATTTCGCTTGCTCTTCTC	
IG-DMR-F1	TAAGTGTTGTGGTTTGTTATGGGTA	
IG-DMR-R1	CCATCCCCTATACTCAAAACATTCT	
IG-DMR-F2	TGGTTTGTTATGGGTAAGTTTTATG	
IG-DMR-R2	CTTCCCTCACTCCAAAAATTAAAA	
Gtl2-DMR-F	AAATTTTGTAAGGAAAAGAATTTTTAGG	Primers for Methylation analysis
Gtl2-DMR-R	TTCAAAATTACTAATCAACATAAACCTC	
